# Time-to-event overall survival prediction in glioblastoma multiforme patients using magnetic resonance imaging radiomics

**DOI:** 10.1007/s11547-023-01725-3

**Published:** 2023-09-26

**Authors:** Ghasem Hajianfar, Atlas Haddadi Avval, Seyyed Ali Hosseini, Mostafa Nazari, Mehrdad Oveisi, Isaac Shiri, Habib Zaidi

**Affiliations:** 1 Division of Nuclear Medicine and Molecular Imaging, Geneva University Hospital, 1211 Geneva, Switzerland; 2 School of Medicine, Mashhad University of Medical Sciences, Mashhad, Iran; 3 Translational Neuroimaging Laboratory, The McGill University Research Centre for Studies in Aging, Douglas Hospital, McGill University, Montréal, QC Canada; 4 Department of Medical Physics, School of Medicine, Shahid Beheshti University of Medical Sciences, Tehran, Iran; 5 Department of Computer Science, University of British Columbia, Vancouver, BC Canada; 6 Geneva University Neurocenter, Geneva University, Geneva, Switzerland; 7 Department of Nuclear Medicine and Molecular Imaging, University of Groningen, University Medical Center Groningen, Groningen, Netherlands; 8 Department of Nuclear Medicine, University of Southern Denmark, Odense, Denmark

**Keywords:** MRI, Glioblastoma, Radiomics, Machine learning, Overall survival

## Abstract

**Purpose:**

Glioblastoma Multiforme (GBM) represents the predominant aggressive primary tumor of the brain with short overall survival (OS) time. We aim to assess the potential of radiomic features in predicting the time-to-event OS of patients with GBM using machine learning (ML) algorithms.

**Materials and methods:**

One hundred nineteen patients with GBM, who had T1-weighted contrast-enhanced and T2-FLAIR MRI sequences, along with clinical data and survival time, were enrolled. Image preprocessing methods included 64 bin discretization, Laplacian of Gaussian (LOG) filters with three Sigma values and eight variations of Wavelet Transform. Images were then segmented, followed by the extraction of 1212 radiomic features. Seven feature selection (FS) methods and six time-to-event ML algorithms were utilized. The combination of preprocessing, FS, and ML algorithms (12 × 7 × 6 = 504 models) was evaluated by multivariate analysis.

**Results:**

Our multivariate analysis showed that the best prognostic FS/ML combinations are the Mutual Information (MI)/Cox Boost, MI/Generalized Linear Model Boosting (GLMB) and MI/Generalized Linear Model Network (GLMN), all of which were done via the LOG (Sigma = 1 mm) preprocessing method (C-index = 0.77). The LOG filter with Sigma = 1 mm preprocessing method, MI, GLMB and GLMN achieved significantly higher C-indices than other preprocessing, FS, and ML methods (all *p* values < 0.05, mean C-indices of 0.65, 0.70, and 0.64, respectively).

**Conclusion:**

ML algorithms are capable of predicting the time-to-event OS of patients using MRI-based radiomic and clinical features. MRI-based radiomics analysis in combination with clinical variables might appear promising in assisting clinicians in the survival prediction of patients with GBM. Further research is needed to establish the applicability of radiomics in the management of GBM in the clinic.

**Supplementary Information:**

The online version contains supplementary material available at 10.1007/s11547-023-01725-3.

## Introduction

Glioblastoma Multiforme (GBM) represents the predominant aggressive tumor of the brain and spinal cord [1]. Unfortunately, this aggressive tumor has a mean survival time of fewer than 15 months. Moreover, its inadequate response to current treatment options and complex progression patterns makes clinical decision-making harder for physicians [2, 3]. Therefore, overall survival (OS) prediction, as a component of prognostication, is a major area of interest in oncological studies. It is evident from the literature that several studies have assessed the predictive power of demographical, clinical, or laboratory data in GBM patient survival analysis. For example, a landmark study by Czipska et al. [4] assessed the prognostic factors of long-term survival for patients with GBM. In another systematic review [5], three types of outcomes for OS prediction, including continuous, binary, and time-to-event, were reported as defined in the reviewed articles.

Despite the decent predictive power of clinical-related features shown by previous studies, most studies intended to examine the prognostication ability of imaging biomarkers [6]. Magnetic Resonance Imaging (MRI) is the most frequent diagnostic procedure utilized in the detection and evaluation of GBM. Radiologists integrate several conventional qualitative and quantitative MRI assessment methods [7, 8]. However, conventional qualitative evaluation might have some limitations and overlook the hidden layers of information within images. In this light, the term “radiomics” can be put forward as it fundamentally describes the analysis of medical images via computational data extraction and, in other words, transforming images into minable biomarkers [9–11]. Radiomics can help us prepare data hidden in images that cannot be seen with conventional image assessment methods with the naked eye in different diseases [12–16]. Whether in the diagnostic or the prognostic area, cancer research has always been of interest to radiomics researchers, and GBM is not an exception [17, 18].

A growing body of research recognizes the efficiency of radiomics analyses for GBM tumors [19, 20]. A recent study by Artzi et al. [21] studied the efficiency of radiomics for differentiating brain metastasis versus GBM. The study of Buchlak et al. [22] is also worth mentioning as they systematically reviewed previous studies focusing on GBM diagnosis via radiomics assessment. In a study by Bae et al. [23], the authors found that adding radiomic features to patients’ genetic and clinical profiles can improve survival prediction. Other studies reached a similar conclusion as MRI-based radiomics and machine learning (ML) algorithms could predict the OS of patients with decent statistics [24–27]. While several studies reported on the use of radiomics in neurodegenerative disorders or GBM, most have utilized ML to binary (high vs. low risk or short vs. long survival time) or multiple (high, intermediate, and low risk or short, intermediate, and long survival time) classification of OS in GBM patients. Therefore, our study's contribution is in using preprocessing methods, feature selectors, and MLs to predict time-to-event survival using MRI-based radiomics features. Therefore, clinicians need to have a more comprehensive knowledge of GBM management by further elaborating on patients’ prognoses. In this study, we aimed to evaluate the applicability of ML algorithms to MRI-based radiomic features along with clinical variables to predict patients’ time-to-event OS.

## Materials and methods

**Fig. 1:**
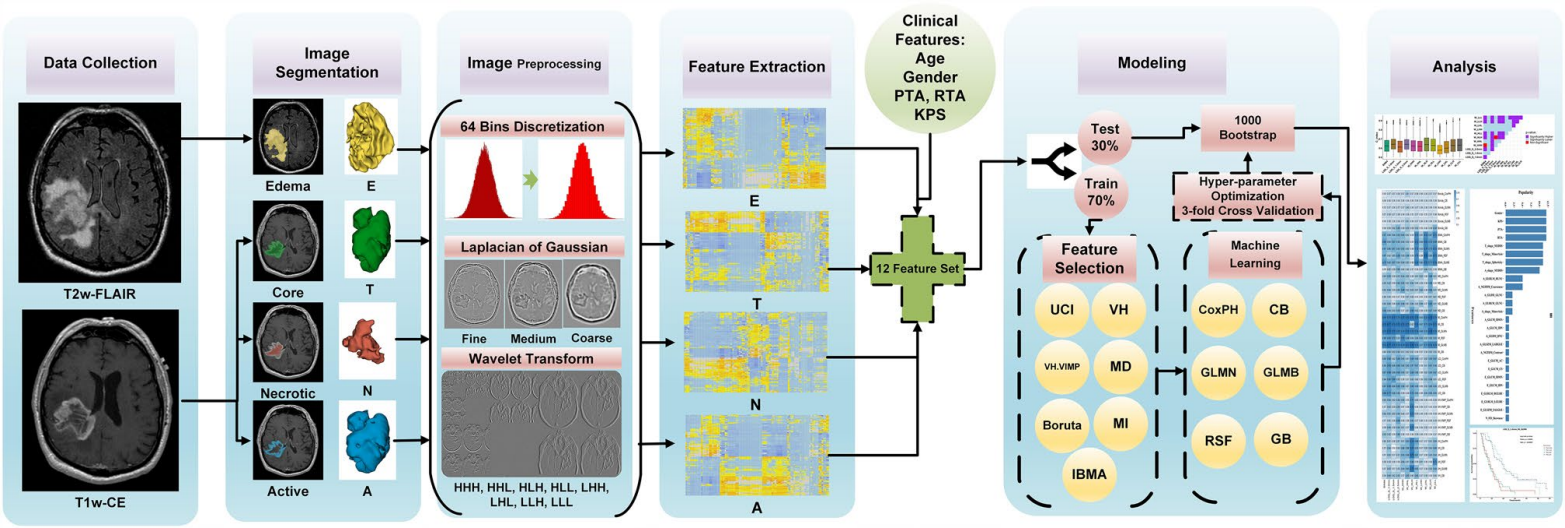
Flowchart of the various steps followed in the present study protocol. Feature selection methods include Univariate C-Index (UCI), Boruta, Variable hunting Variable Importance (VH.VIMP), Variable hunting (VH), Minimal Depth (MD), Mutual Information (MI), and Iterated Bayesian Model Averaging (IBMA). Machine learning methods include Cox Proportional Hazard regression (CoxPH), Cox Boost (CB), Generalized Linear Model Network (GLMN), Random Survival Forest (RSF), GLM Boosting (GLMB), and Gradient Boosting (GB)

Figure 1 illustrates a flowchart of the various steps followed in the present study protocol.

### Data and image acquisition

**Fig. 2:**
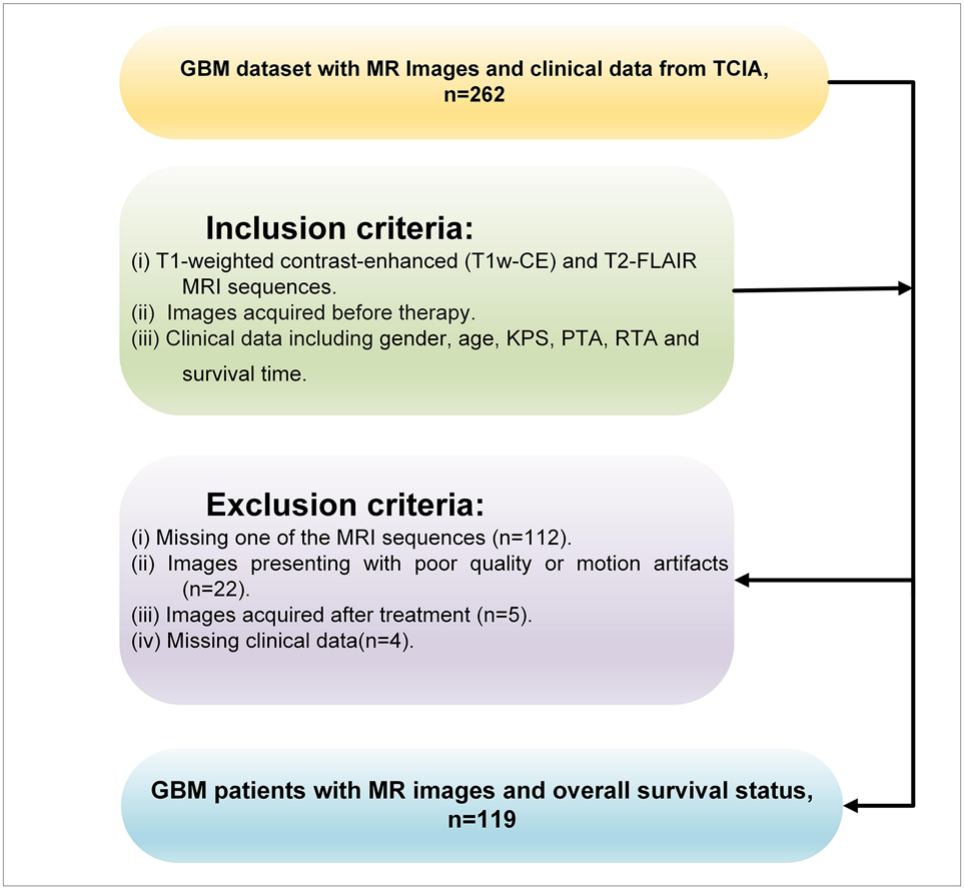
Flowchart of inclusion and exclusion criteria in this study. Karnofsky Performance Score (KPS), radiation treatment adjuvant (RTA), pharmaceutical treatment adjuvant (PTA)

The Cancer Imaging Archive (TCIA) was used to download images and clinical information of the enrolled patients [28, 29]. The following were the inclusion criteria for enrolment in this study protocol: (1) All GBM patients who underwent T1-weighted contrast-enhanced (T1w-CE) and T2-FLAIR MRI sequences AND (2) Images acquired before therapy AND (3) All patients have clinical data including gender, age, Karnofsky Performance Score (KPS), radiation treatment adjuvant (RTA), pharmaceutical treatment adjuvant (PTA), and survival time with follow-up event status (expired and alive). The exclusion criteria for this study include: (1) Missing one of the MRI sequences; (2) Images presenting with poor quality or motion artifacts; (3) Images acquired after treatment, and (4) Missing clinical data. Figure 2 shows a flowchart of inclusion and exclusion criteria.

After applying inclusion and exclusion criteria, 119 GBM patients with T1w-CE and T2-FLAIR MRI sequences, clinical data, and survival time were enrolled in this study protocol. MR image sequence specifications are summarized in Table 1. Patients collected clinical and demographical information, including gender, age, MGMT status, Karnofsky Performance Score (KPS), tumor histology subtype, RTA, and PTA used as clinical variables and time-to-event OS as a target. Due to the unavailability of the MGMT status and tumor histology subtype variables, these variables were removed from this study. KPS is an index reflecting the functional performance of cancer patients with 11 levels ranging from 0 (expired) to 100 (normal) [30]. The clinical and demographic data of patients are shown in Table 2.

**Table 1:** Acquisition parameters of MR image sequences used in this study

Image	Flip angle (°)	Echo time (ms)	Repetition time (ms)		Inversion time (ms)	Manufacturer	Magnetic field strength
T1w-CE	60–90	10.88 ± 3.45		1010 ± 960	–	GE MEDICAL SYSTEMS, SIEMENS, PHILIPS MEDICAL SYSTEMS	1.5 T, 3.0 T
T2w-FLAIR	90–180	132.36 ± 18.3		9730 ± 1544	2352 ± 217		

*ms* milliseconds

**Table 2:** Characteristics of GBM patients included in this study

Characteristic	Number of patients (*N* = 119)
*Gender*	
Male	73 (61.34%)
Female	46 (38.66%)
Age (year) ± SD	57.83 ± 13.65
*MGMT Status*	
Methylated	45 (37.82%)
Unmethylated	37 (31.09%)
Not available	37 (31.09%)
*Karnofsky Performance Score*	
≤ 70	86 (72.27%)
> 70	33 (27.73%)
Tumor histology subtype	55 (46.22%)
Available	55 (46.22%)
Classical	11
Mesenchymal	21
Neural	8
Proneural	15
Not available	64 (53.78%)
*Radiation Treatment Adjuvant*	
Yes	103 (86.55%)
No	16 (13.45%)
*Pharmaceutical Treatment Adjuvant*	
Yes	105 (88.24%)
No	14 (11.76%)
*Therapy*	
Radiation and Pharmaceutical adjuvant	93 (78.15%)
Only Radiation adjuvant	10 (8.40%)
Only Pharmaceutical adjuvant	12 (10.08%)
No Treatment	4 (3.37%)
*Survival status in months*	
All patient’s mean ± SD (median)	14.37 ± 12.85 (10.35)
Expired # (mean ± SD)	92 (13.42 ± 11.91)
Alive # (mean ± SD)	27 (17.60 ± 15.45)

*MGMT* O-6-methylguanine-DNA methyltransferase

### Image preprocessing

A 64 bin discretization, Laplacian of Gaussian (LOG), and Wavelet Transform were used to preprocess MR images prior to feature extraction to achieve multiple sets of image features. Sigma values of 1, 1.5, and 2.5 mm were utilized for applying the LOG filter as fine, medium, and coarse filters, respectively [31]. The Wavelet Transform was performed via eight distinct decompositions, including LLL, LLH, LHL, LHH, HLL, HLH, HHL, and HHH, in which L and H stand for low-pass and high-pass filters, respectively, applied to the three-dimensional region. Overall, we utilized 12 distinct preprocessing methods [31].

### Segmentation

Following the aforementioned preprocessing methods and using the 3D Slicer version 4.9 [32], we manually delineated and segmented four volumes of interest (VOI) for each patient, including core tumor (VOI_T_), active enhanced tumor (VOI_A_) and necrotic tumor (VOI_N_) regions from T1w-CE and edema (VOI_E_) region from T2-FLAIR. VOIs. Manual segmentation was performed by two experienced medical physicists with 4–6 years of experience. In addition, the manual segmentation was visually inspected and manually corrected, when necessary, by an experienced radiologist with 8 years of experience. The corrected VOIs were then used in subsequent analyses.

### Feature extraction

Three feature types were extracted from each of the four VOIs, including shape features (*n* = 13), first-order (FO) features (*n* = 18), and texture features (*n* = 74) (Gray Level Size Zone Matrix (GLSZM), Gray Level Co-occurrence Matrix (GLCM), Neighboring Gray Tone Difference Matrix (NGTDM), Gray Level Run Length Matrix (GLRM), and Gray Level Dependence Matrix (GLDM)). For each preprocessing method, 92 features, including FO and texture features, were extracted (shape features were the same for all preprocessing methods). The whole tumor was also used to calculate three shape features, including A/T, N/T, and T/E volume ratios. In total, 4471 features ([92 × 12 × 4] + [13 × 4] + 3) were extracted from 4 VOIs and 12 image preprocessing methods. Radiomic features were extracted by the Pyradiomics python library [33], which is compliant with IBSI guidelines [31, 34].

### Feature selection

A careful selection process took place, during which a group of extracted features were chosen with the assistance of various feature selection (FS) algorithms. The seven FS methods used in this study included Univariate C-Index (UCI), Boruta, Variable hunting Variable Importance (VH.VIMP), Variable hunting (VH), Minimal Depth (MD), Mutual Information (MI), and Iterated Bayesian Model Averaging (IBMA). Further details about these FS methods are provided in the Supplementary section.

### Machine learning models

The preprocessing procedures and FS methods along with six ML models (Cox Proportional Hazard regression (CoxPH), Cox Boost (CB), Generalized Linear Model Network (GLMN), Random Survival Forest (RSF), GLM Boosting (GLMB), and Gradient Boosting (GB)) were evaluated by multivariate analysis using the C-index [35]. More details about the ML methods are provided in the Supplementary section.

### Modeling and statistical analysis

In the first step, data were split into training (70%) and test (30%) datasets. Subsequently, FS methods were implemented on the training dataset, and the selected features were fed to ML models. Next, hyperparameter optimization with grid search and threefold cross-validation (fivefold cross-validation in supplemental data) was performed for ML models in the training dataset. The details of hyperparameter optimization are shown in Table 3. 1000 bootstraps were performed on the testing dataset to assess the models, and mean ± standard deviation (SD) and 95% confidence interval (95% CI) were reported for each model.

**Table 3:** Details of hyperparameters optimization

Model	R package	Hyperparameter
Coxph	Survival	–
CB	CoxBoost	maxstepno: 50–500
GLMN	glmnet	s: 0.001–0.1 alpha: 0–1
RSF	randomForestSRC	split-rule: logrank, logrankscore mtry: 1–10 nodesize: 1:20 ntree: 100, 500, 1000
GLMB	mboost	mstop: 50–500
GB	gbm	shrinkage: 0.01,0.05,0.1 interaction.depth: 1–5 n.trees: 100, 500, 1000 n.minobsinnode: 3–5

We also performed the Wilcoxon rank sum test among different preprocessing, FS, and ML algorithms and reported the results as box plots and *p* value tile plots. Kaplan–Meier of the best models with a log-rank p value for the training and test datasets was drawn. A *p* value of 0.05 was considered as the cut-off for statistically significant differences. Modeling and statistical analysis were implemented in R version 4.0 (R Foundation for Statistical Computing, Vienna, Austria) [36].

## Results

### Characteristics of GBM patients

In this study, we used data from 119 GBM patients with a mean age (± SD) of 57.83 (± 13.65), with 73 (61.34%) of them being male. Nighty-two patients (77.3%) with a mean survival (± SD) of 13.42 ± 11.91 months passed, while 27 patients (22.7%) with a mean survival (± SD) (i.e., follow-up time) of 17.60 ± 15.45 were alive and considered as censor data.

### Feature selection analysis

**Fig. 3:**
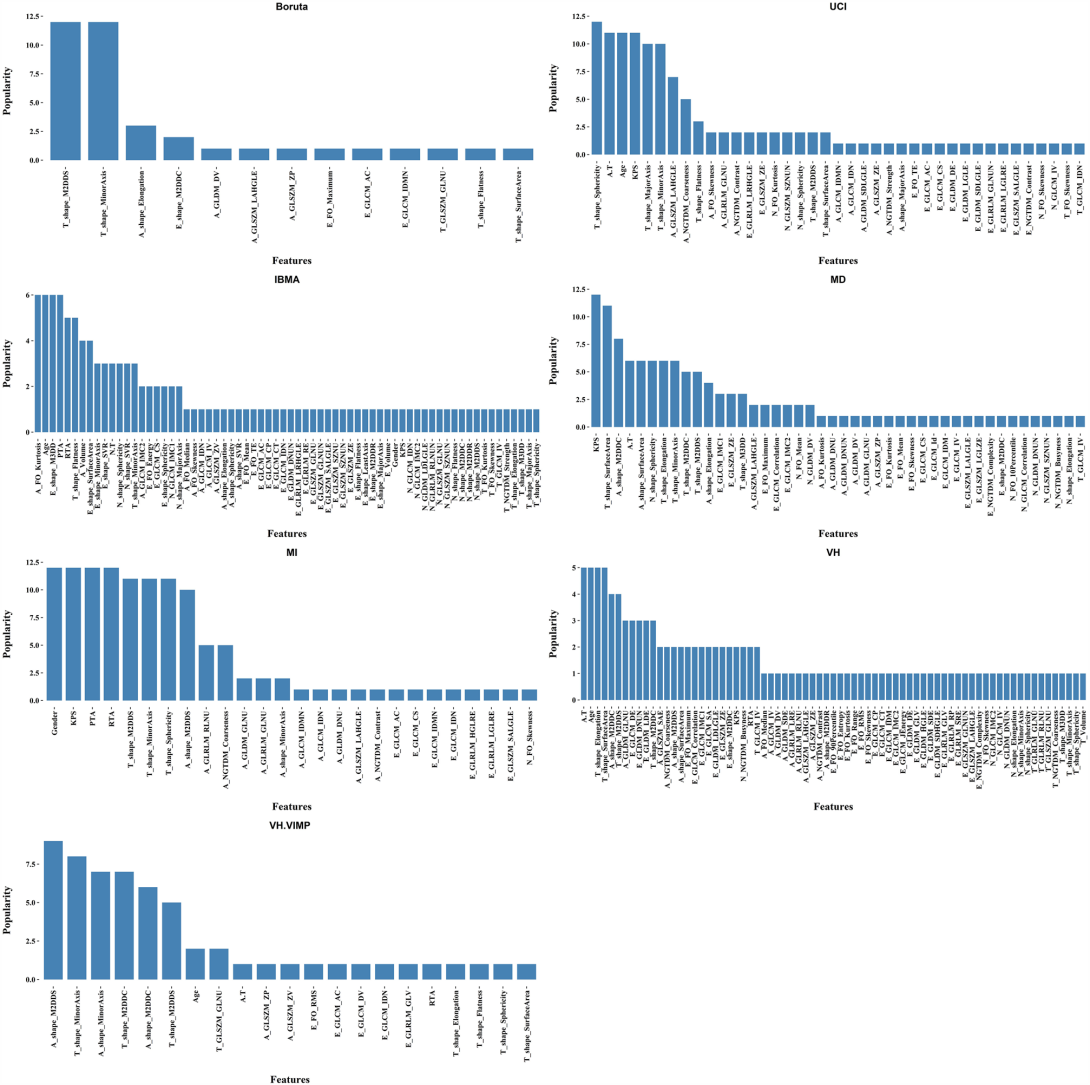
The list of features selected by Boruta, Univariate C-Index (UCI), Iterated Bayesian Model Averaging (IBMA), Minimal Depth (MD), Mutual Information (MI), Variable hunting Variable Importance (VH.VIMP), and Variable hunting (VH)

**Fig. 4:**
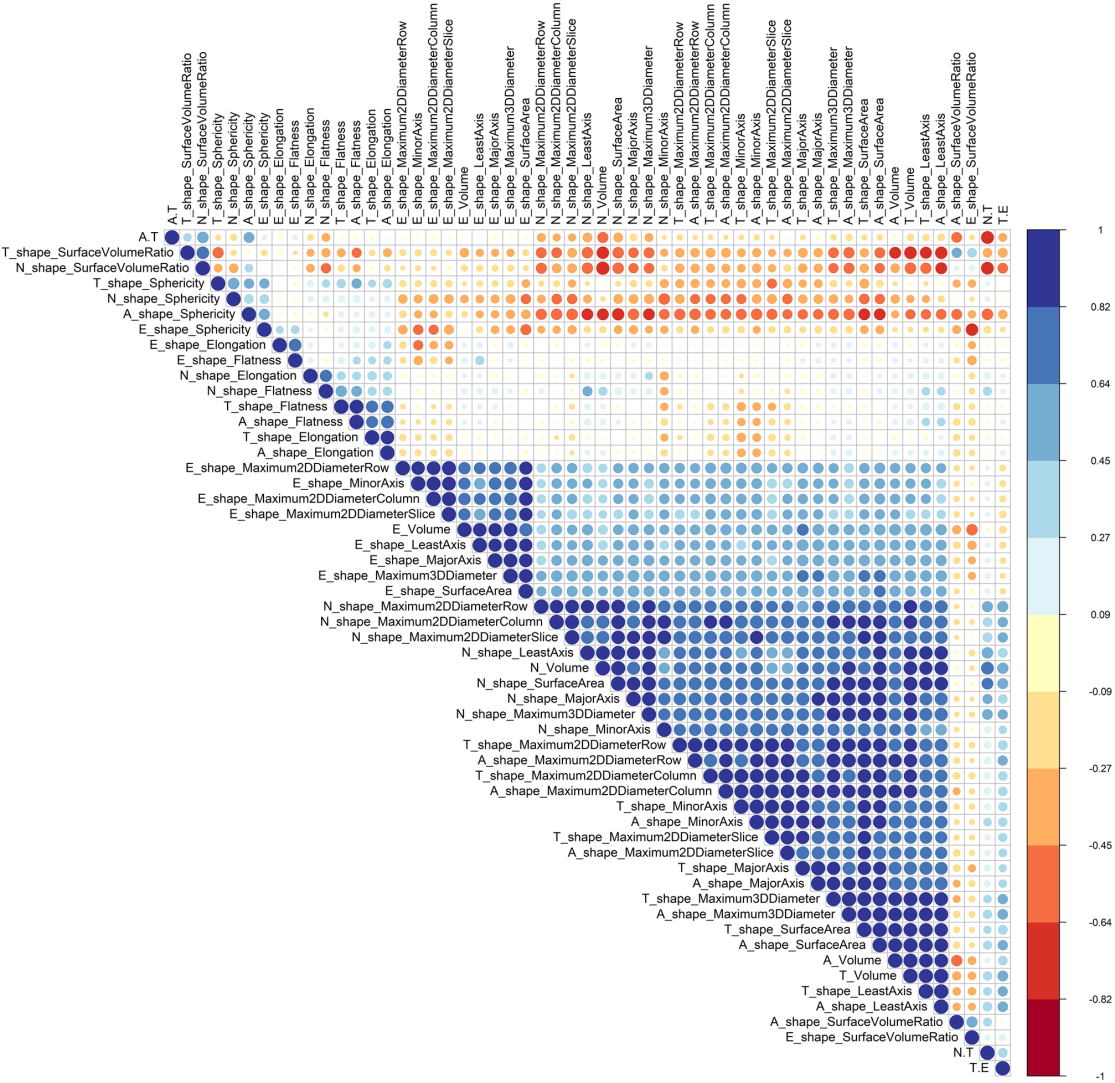
Spearman correlation coefficients of radiomic shape features

The feature selectors had decent power for selecting the most important features related to our study purpose. The popularity of the selected features for each FS method can be found in Fig. 3. Our results showed that seven FS methods, including Boruta, UCI, IBMA, MD, MI, VH, and VH.VIMP selected and ranked 38, 120, 120, 120, 120, 120, and 59 features, respectively. Among the features which were selected, more than 10 times out of 697 features (151 unique features), MinorAxis (*n* = 51), Maximum 2D diameter slice (M2DDS) (*n* = 39), Sphericity (*n* = 26), SurfaceArea (*n* = 20), Maximum 2D diameter column (M2DDC) (*n* = 15) and Elongation (*n* = 13) from shape features of VOI_T_, KPS (*n* = 38), age (*n* = 24), RTA (n = 20), PTA (*n* = 18) and gender (*n* = 13) from clinical features, M2DDS (*n* = 21), M2DDC (*n* = 18), MinorAxis (*n* = 17), SurfaceArea (*n* = 13) and A/T ratio (n = 16) from shape features of VOI_A_, and NGTDM_Coarseness (*n* = 12) and GLSZM_LAHGLE (*n* = 12) from texture features of VOI_A_ were repeatedly selected by most of the seven FS algorithms. Figure 4 shows Spearman’s correlation coefficient of shape features.

### Model analysis

**Fig. 5:**
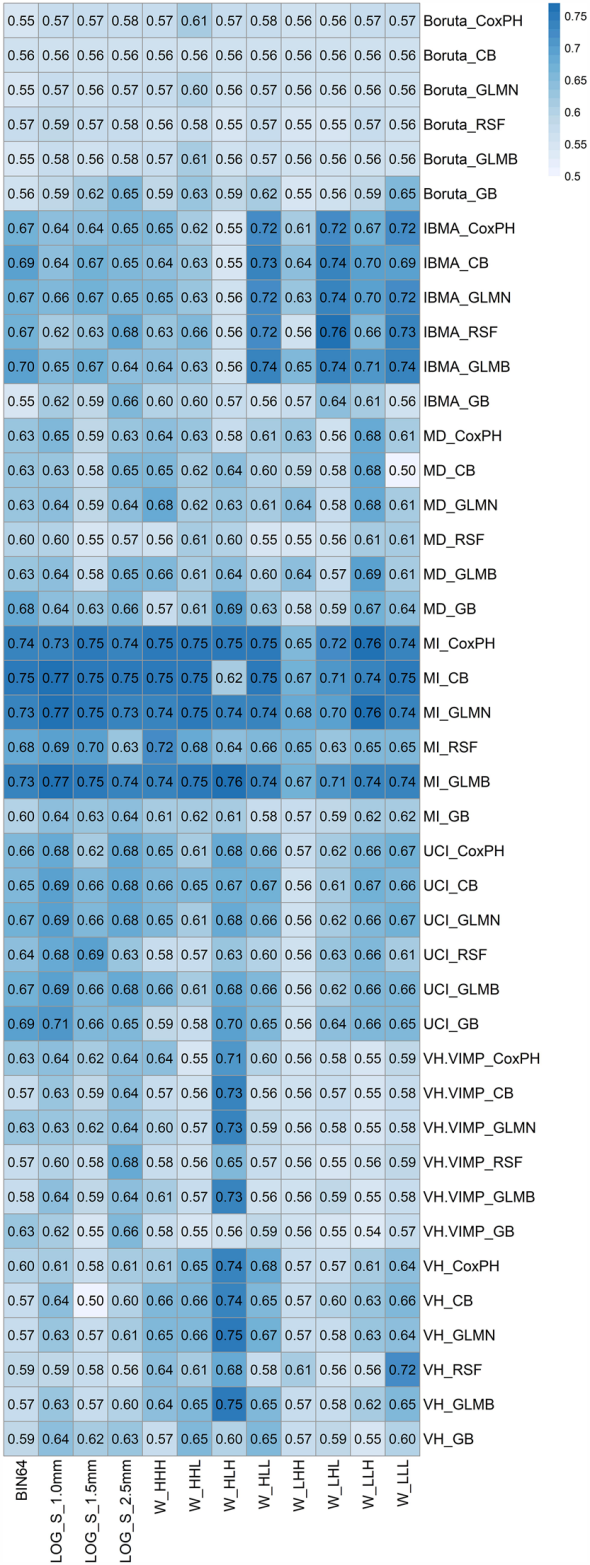
Heat map of C-indices for each model combining preprocessing methods, features selection, and machine learning algorithms (504 models in total)

**Fig. 6:**
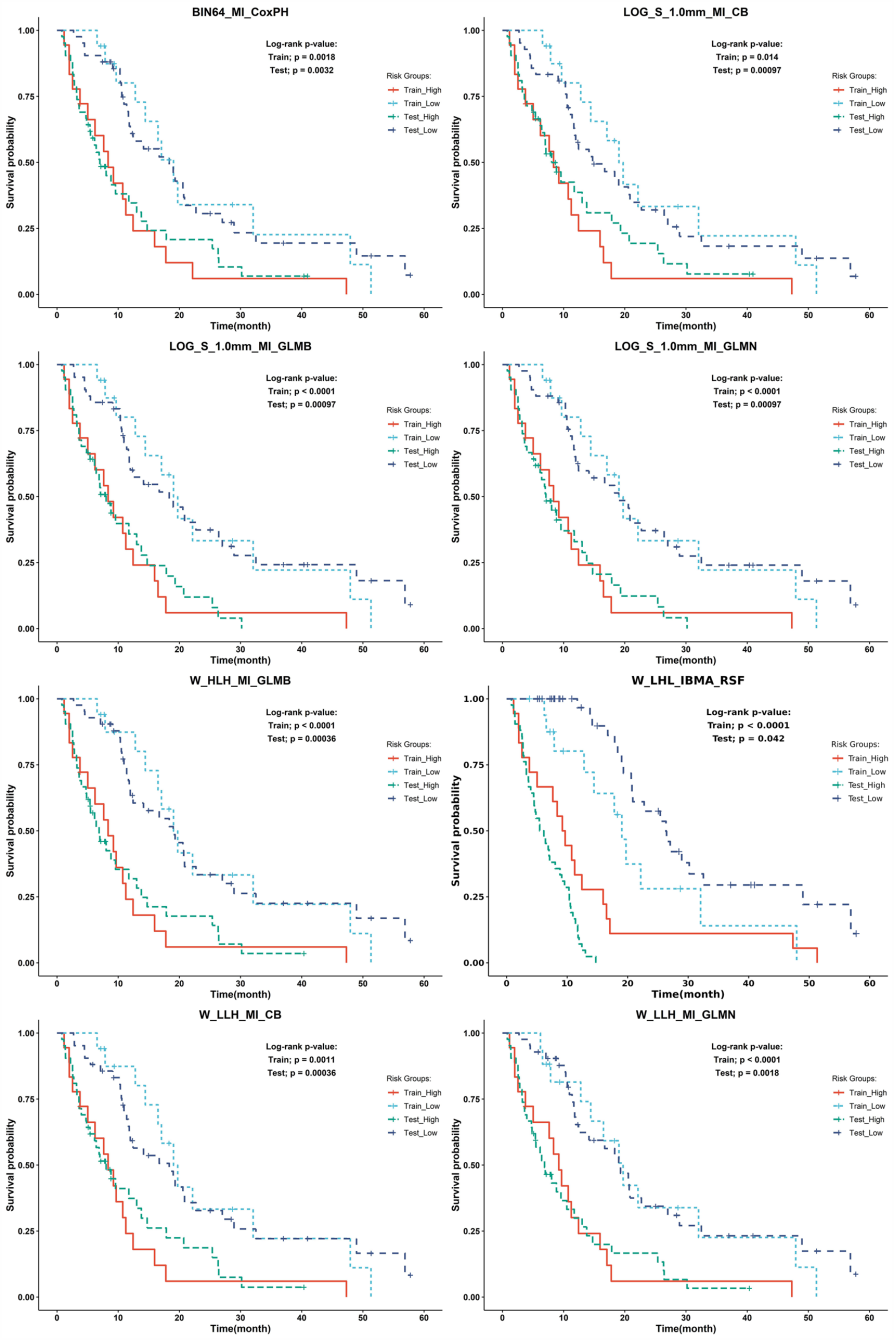
Kaplan–Meier curves corresponding to the eight best models with their corresponding log-rank p value in the train and test datasets

A heat map of mean C-indices of 1000 bootstraps for each model is shown in Fig. 5. We also generated a heat map of 95% CI and mean ± SD in supplemental Figs. 1S and 2S, respectively. We also generated a heat map of the mean C-indices of these models with fivefold cross-validation for hyperparameter optimization in Fig. 3S. A difference heat map of threefold cross-validation with respect to fivefold cross-validation C-indices for each model is depicted in Fig. 4S. It can be seen that the range of C-index difference is 0 to 0.25 (less than 0.1 in most algorithms) and that the threefold cross-validation for hyperparameter optimization is slightly better on most of the models. As such, the rest of the analysis of this study is based on a threefold cross-validation scheme. Our multivariate model analysis showed that the best prognostic FS/ML combination is the MI/CB applied to the LOG (Sigma = 1 mm) preprocessing method (C-index = 0.77 ± 0.05) (mean ± SD), 0.77–0.77 (95% CI)). The second best-performing combinations were MI/GLMB and MI/GLMN applied to the LOG (Sigma = 1 mm) preprocessing method (C-index = 0.77 ± 0.05, 0.76–0.77). Figure 6 shows Kaplan–Meier curves of the eight best models. All these models had significant log-rank p values in the training and testing datasets.

### Statistical analysis

**Fig. 7:**
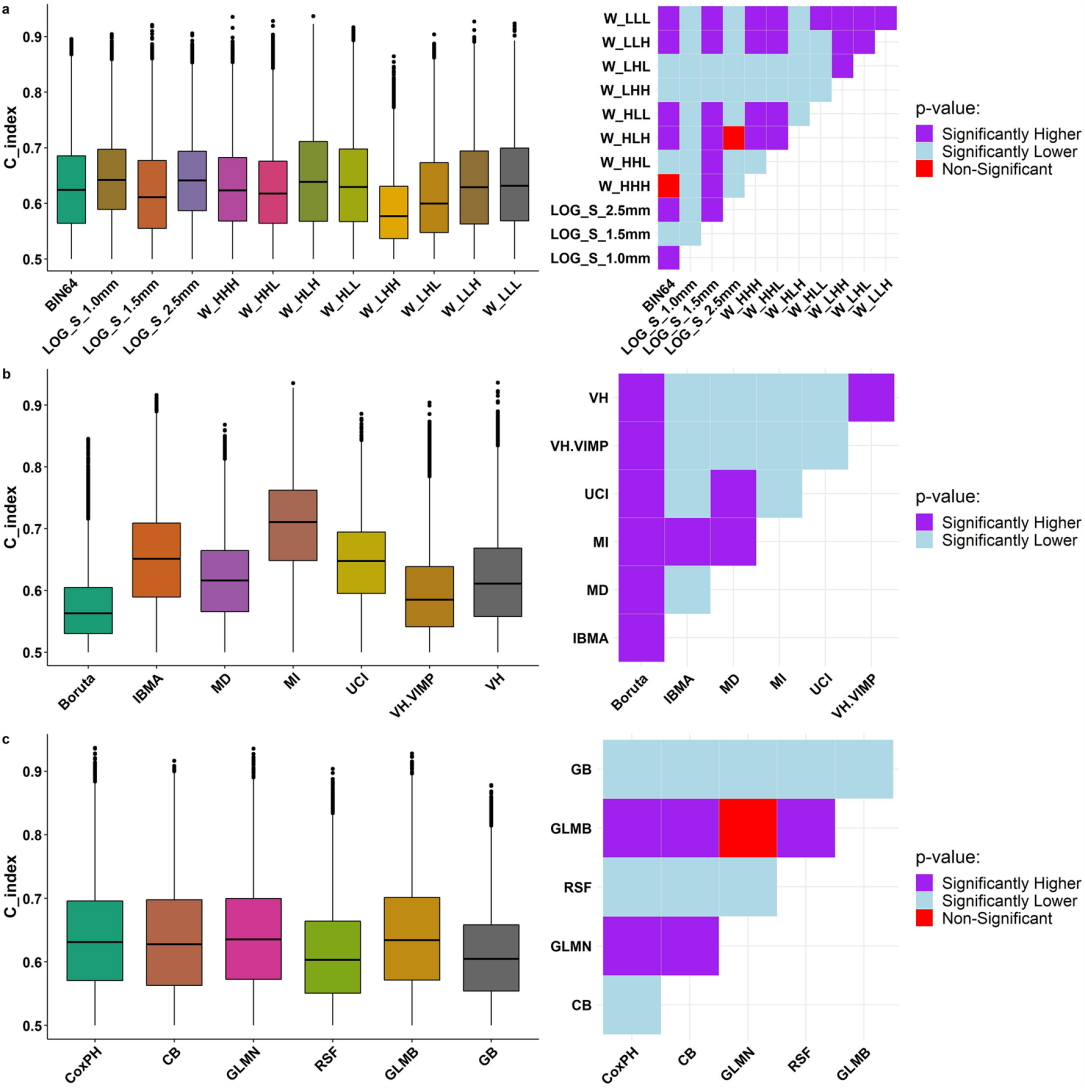
Comparison of different preprocessing (**a**), features selection (**b**), and machine learning (**c**) methods in box plots (left) and Wilcoxon *p* value tile plots (right)

A comparison of the different preprocessing, FS, and ML methods are depicted in Fig. 7 as box plots (left) and Wilcoxon *p* value tile plots (right). For preprocessing methods (Fig. 7a), the LOG filter with Sigma of 1.0 mm had a higher significant *p* value (mean C-index = 0.65) than other methods. Wavelet transforms (HLH form) and LOG filter with Sigma of 2.5 with a mean C-index of 0.64 had a higher significant *p* value than other preprocessing methods (except LOG filter with Sigma = 1.0 mm). Figure 7b indicates that MI performed better (mean C-index = 0.70) than other FS methods. In the second place, the IBMA method, with a mean C-index of 0.65, performed better than other FS methods except for the MI method. Figure 7c demonstrates that the GLMB, and GLMN model with a mean C-index of 0.64 had a higher significant *p* value than other models. CoxPH is the second best model (mean C-index = 0.64) with a significantly higher *p* value than other models, except the GLMB and GLMN models.

## Discussion

Our study demonstrated that ML algorithms are capable of predicting the time-to-event OS of patients through the use of MRI-based radiomic features. In an extensive systematic review, the 2-year OS of patients was comprehensively reviewed and analyzed considering a large number of articles [37]. It has been reported that the 2-year and 3-year OS of GBM patients is 18% and 11%, respectively. Other clinical trials indicated a 2-year survival of 26–33% and an approximate median OS of 15 months [37, 38]. Another study reported that patients with confirmed GBM had a 5.6% 5-year survival rate [1]. In our study, 77% of the patients decreased after 13.42 ± 11.91 (mean ± SD) months, while 23% were in the survivor group after 17.60 ± 15.45 months. We used seven distinct feature selectors and six ML methods applied to radiomic features derived from MR images preprocessed with twelve different methods. This leads to 504 models, the performance of which was assessed using the C-index. Many research and development efforts focused on assessing OS in GBM patients. The best model was the MI/CB applied to the LOG (Sigma = 1 mm) preprocessing method (C-index = 0.77 ± 0.05) (mean ± SD), 0.77–0.77 (95% CI)). LOG with Sigma = 1 mm, also known as the fine filter, had better performance, significantly higher than other methods. Among FS methods, MI had the best performance. Conversely, the GLMB, and GLMN models had significantly better performance than other ML algorithms.

Regarding the use of radiomics for OS prediction, the models perform decently and satisfactorily, according to the latest studies. Bakas et al. [26] used MRI-based radiomic features to predict survival status with 74.26% accuracy. In another study, it was claimed that PET-based radiomic features could contribute to the prognosis and indicate whether patients benefit from re-irradiation (*p* value for OS prediction < 0.05) [44]. Jajroudi et al. [39] examined the ability of MRI-extracted radiomic features to determine GBM survival. The mean percentage of sensitivity, accuracy, AUC, and specificity of the four implemented ML classifier was 82.54, 80.47, 85, and 79.78, respectively. In a retrospective ML/radiomics study, Cepeda et al. [40] reported that RSF achieved an integrated AUC (iAUC) of 0.76 and a C-Index of 0.61 with six months follow-up. Moreover, Chaddad et al. [41] found a decent predictive value of radiomics signature for determining patients’ survival time with an AUC of 78.56%. In addition, the radiomic features proved to be reliable and repeatable among different centers and datasets [45]. To estimate the survival time in GBM patients, Hedyehzadeh et al. [42] studied a dataset of 118 GBM patients. They computed C-index using eight traditional and three convolutional neural networks (CNN)-based regression techniques to predict survival periods. GoogleNet achieved the highest performance with a C-index of 0.89.

As mentioned earlier, we used only radiomic features and clinical variables for OS prediction. Similar to our approach, Ammari et al. [24] utilized radiomics and clinical data to stratify patients based on their survival. Using ML regression algorithms, they achieved a C-index of 0.64 for the prediction of OS. Although these decent results arise from relatively comprehensive models (radiomics + clinical and/or genomic data), our study could reach fair prediction accuracies using radiomics + clinical data. Chen et al. [43] showed that radiomics-only models performed better than clinical-only models. In our study, the mean C-index of the combination of preprocessing/FS/ML algorithms was in the range of 0.50–0.77 in the test dataset.

Nevertheless, several previous studies combined radiomics with features other than clinical for model construction. For example, a study by Bae et al. [23] extracted radiomic features and constructed a model using clinical, genetic, and radiomic features. They compared the model’s performance with another one consisting of clinical and genetic data and reported promising results demonstrating that adding radiomic features can significantly improve the OS and progression-free survival (PFS) prediction power of ML models with *p* values of 0.04 and 0.03, respectively. In addition, the iAUC of their model plus RSF algorithm was 0.65 (radiomics only) and 0.76 (radiomics + clinical) for OS prediction.

We compared the latest studies to ours in Table 4. These studies used random split and cross-validation for the validation of their models. None of these studies used external validation dataset. These studies used two types of models, including classification and time-to-event OS prediction. In our study, we used random split for model validation. We also used cross-validation for hyperparameter optimization and bootstrap on the testing dataset. While we used time-to-event OS prediction, the results of our best model is comparable with these studies.

**Table 4:** Comparison of recent similar studies with our study

Study	Patient size	Type	Metric	Validation
Bakas et al. [26]	101	Classification	ACC = 0.74	Fivefold cross-validation
Jajroudi et al. [39]	55	Classification	AUC = 0.85, ACC = 0.80, SEN = 0.82, SPE = 0.80	Tenfold cross-validation
Cepeda et al. [40]	203	Time-to-event	C-index = 0.61 iAUC = 0.76	Random Split
Chaddad et al. [41]	73	Classification	AUC = 0.76	Fivefold cross-validation
Hedyehzadeh et al. [42]	118	Time-to-event Regression	C-index = 0.89	Fivefold cross-validation
Chen et al. [43]	127	Classification	AUC = 0.81 HR = 3.65	Random Split
Bae et al. [23]	217	Time-to-event	iAUC (Radiomics) = 0.65 iAUC (Clinical + Radiomics) = 0.760	Random Split
Ammari et al. [24]	194	Time-to-event	C-index = 0.64	Random Split
Our Study	119	Time-to-event	C-index = 0.77	Random Split 1000 Bootstrap on test data

*ACC* accuracy, *AUC* area under the curve, *HR* hazard ratio, *integratedAUC* iAUC, *SEN* sensitivity, *SPE* specificity

We had four feature types for overall survival prediction in GBM patients. The number (percent) of clinical, shape, FO, and texture features selected by feature selectors were 113 (16%), 366 (53%), 43 (6%), and 175 (25%), respectively, more than half of which belonged to the shape category. We also had four VOIs for feature extraction. The number (percent) of features selected by feature selectors in VOI_T_, VOI_A_, VOI_N_, and VOI_E_ was 207 (37%), 156 (28%), 54 (10%), and 141 (25%), respectively. The results indicated that core tumor (VOI_T_) in GBM patients greatly impacted survival prediction. MinorAxis from VOI_T_ was the most repeated feature among the top selected features. Based on IBSI guidelines [34, 46], MajorAxis, MinorAxis, LeastAxis, Flatness, and Elongation are computed by principal component analysis (PCA) of VOI. MajorAxis, MinorAxis, and LeastAxis are the largest, second largest, and smallest eigenvalues, respectively, calculated by PCA [34, 46]. Flatness is the ratio of LeastAxis to MajorAxis, whereas Elongation is the ratio of MinorAxis to MajorAxis. In several studies [47, 48], MajorAxis had a significant log-rank *p* value. In our study, this feature was selected by UCI and IBMA feature selection techniques. While MinorAxis had no significant log-rank p value in a previous study [48], our study selected all feature selectors to cross several preprocessing sets. M2DDS of VOI_T_ is the maximal diameter on the axial plane [34, 46], equivalent to Response Evaluation Criteria in Solid Tumors (RECIST) [49]. Galanis et al. [50] showed that this feature could predict OS, while our study selected 6 FSs. The sphericity of VOI_T_ is another top-selected feature. This feature measured how the VOI is sphere-like [34, 46]. In Sanghani et al. [51] study, sphericity was predictive of OS prognosis. In another study [52], sphericity or surface regularity and age were predictors of overall survival, but the surface area had no significant *p* value. Figure 4 indicates that the shape feature of VOI_T_ and VOI_A_ had a Spearman’s coefficient of over 0.90. MinorAxis, M2DDS, M2DDC, and SurfaceArea from VOI_A_ had the same interpretation as VOI_T_ features. The Coarseness feature of NGTDM from VOI_A_ implies that on the spatial rate of change, that lower value shows a higher spatial change rate and non-uniform texture [34, 46]. This pattern is an enhanced area of the tumor correlates with lower OS in GBM patients. Large Area High Gray Level Emphasis (LAHGLE) of GLSZM from VOI_A_ was selected by 5 FSs. This feature emphasizes the high gray level with a large size zone [34, 46]. Larger values indicate lower OS, and those patients are at high risk. A/T volume ratio is the proportion of contrast-enhanced tumors. This feature was selected by 4 FSs and associated with OS in several studies [47, 48, 53]. In the clinical features, KPS and age were selected by 5 and 4 FSs, respectively. Gender was selected by MI and IBMA FS methods. Various studies reported that age and KPS were predictors for OS, but gender was not an OS predictor [54–56]. The PTA and RTA are two important variables that demonstrate how patients were treated. The RTA was selected by four FS methods, while the PTA was selected by MI and IBMA FS techniques. These two variables were selected by the MI method across all 12 feature sets, and this FS method outperformed all other strategies. A number of studies reported that using PTA and RTA with total resection improves survival in GBM patients [57–59]. As shown in Table 2, 93 (78%) of GBM patients received both treatments, while 22 (18%) received at least one treatment. Only four patients did not receive treatment.

Our study is not exempt from limitations. First of all, the nature of our study is retrospective, and as such, it is inherently prone to some bias. Secondly, we had no external validation, and our sample size was insufficient to expand our results' generalizability. To overcome this limitation, we used threefold cross-validation for hyperparameter optimization and 1000 bootstrap on the testing dataset. Last, we did not include genetic variables and tumor histology subtype in our modeling to compare it with radiomic features predictions.

## Conclusion

This study showed that the radiomics framework plus clinical data could be utilized in the management of GBM patients. MRI-extracted radiomic and clinical features could predict the patient's time-to-event OS by applying feature selection methods and ML algorithms. MRI-based radiomics combined with clinical variables might be promising in terms of assisting clinicians in the survival prediction of patients with GBM.

### Supplementary Information

Below is the link to the electronic supplementary material.

Supplementary file 1

## Abbreviations

CoxPH: Cox Proportional Hazard regression
CB: Cox Boost
FS: Feature Selection
GBM: Glioblastoma Multiforme
GB: Gradient Boosting
GLMB: Generalized Linear Model Boosting
GLMN: Generalized Linear Model Network
IBMA: Iterated Bayesian Model Averaging
LOG: Laplacian of Gaussian
MD: Minimal Depth
MI: Mutual Information
ML: Machine Learning
MRI: Magnetic Resonance Imaging
OS: Overall Survival
RSF: Random Survival Forest
TCIA: The Cancer Imaging Archive
UCI: Univariate C-index
VH: Variable Hunting
VH.VIMP: Variable Hunting Variable Importance
VOI: Volume of Interest
