# Supplementary material for: Time-to-event overall survival prediction in glioblastoma multiforme patients using magnetic resonance imaging radiomics
Source: Radiol Med. 2023 Sep 26;128(12):1521–34. doi: 10.1007/s11547-023-01725-3 (PMC10700216; doi:10.1007/s11547-023-01725-3)
Supplement: Supplementary file 1 [file 11547_2023_1725_MOESM1_ESM.docx]

**Supplemental Data**

- **Feature Selection explanation**

***Univariate C-index (UCI)***

In the (a) UCI method, the Spearman correlation test is performed at first and the redundant features (R^2^>0.90) will be removed for further analysis. Thereafter, Concordance Index (C-index) is calculated for each feature by 100 bootstrapping in the training dataset. Subsequently, features are sorted by their mean C-indices, the top ten features are selected.

***Minimal Depth (MD), Variable hunting (VH), Variable hunting Variable Importance (VH.VIMP)***

MD, VH, and VH.VIMP are model-based FS methods that use Random Survival Forest (RSF) algorithm ([1](#_ENREF_1), [2](#_ENREF_2)). MD sorts the features based on depth; the closer to the root node, the more predictive power is indicated. The top 10 features with MD are selected. In the VH method, the dataset is randomly split into training and testing datasets, followed by the application of RSF to the training dataset and the selection of random features via M-threshold. The initial model is created by selected features and adding features to the model will continue until the joint variable importance is stabilized. This procedure is repeated 50 times and features with the most frequency are selected. VH.VIMP functions similarly to the VH method except it uses variable importance instead of minimum depth to order the features, which is a faster method.

***Boruta***

Boruta is a wrapper algorithm that uses RSF as a model and creates shadow features by a random shuffle of entire features in which each feature has a shadow feature with random values. The loss accuracy and Z-score are calculated for each feature and corresponding shadow. The criteria for feature selection is the maximum Z-score among shadow attributes. This procedure runs on the RSF model with 100 bootstrap repeats until the entire feature importance is determined. After 100 bootstrapping, the feature with a Z-score significantly higher than the maximum shadow feature is selected (p-value<0.05) ([3](#_ENREF_3)).

***Mutual Information (MI)***

MI has a completely parallelized performance. A linear approximation established on correlation is used to figure MI between two columns. Pearson's or Spearman's estimators are utilized to figure the correlation between continuous variables, whereas Somers' Dxy index is utilized to calculate the correlation between survival data ([4](#_ENREF_4)).

***Iterated Bayesian Model Averaging (IBMA)***

The IBMA approach operates by repeatedly invoking a Bayesian model averaging method and iterating over the variables in a predetermined sequence. Exclusively, those variables with posterior probabilities larger than a defined threshold are preserved after each call for the Bayesian model averaging algorithm; those posterior probabilities that do not match the threshold are replaced with the subsequent group of variables. The order in which the variables are to be examined is generally decided based on a univariate measure of goodness of fit for each variable. By averaging across the best models in the model class according to estimated posterior model probability, Bayesian Model Averaging evaluates all models and accounts for the model uncertainty inherent in the variable selection issue ([5](#_ENREF_5)).

- **Machine Learning models explanation**

***Cox Proportional Hazard regression (CoxPH):***

The hazard function is calculated by the Coxph model using the following equation:

$$h\left( t;X_{1},X_{2},\ldots X_{p} \right)=h_{0}\left( t \right) exp(\beta_{1}X_{1}+\ldots+\beta_{p}X_{p})$$

wherein $h_{0}(t)$ represents the baseline hazard function, $X_{j}$ denotes the covariates (features), and $\beta_{j}$ denotes the coefficients ([6](#_ENREF_6)). The Cox partial likelihood function is maximized to approximate the vector of coefficients:

$$L\left( \beta\right)= \prod_{i=1\ldots n S.t. \delta_{i}=1} \frac{\exp(\beta^{T}X^{i})}{\sum_{l\in R_{i}} {exp(\beta^{T}X^{l})}^{'}}$$

***Cox Boosting (CB)****:*

Component-wise probability-based boosting is used to fit a Coxph model. The loss function for CB is CoxPH and partial likelihood estimation was used for penalizing the model in each boosting step ([7](#_ENREF_7)).

***Generalized Linear Model Network (GLMN):***

Fits a penalized maximum likelihood model with a generalized linear model ([8](#_ENREF_8)).

***GLM Boosting (GLMB):***

A boosting approach based on component-wise univariate linear models is used to fit a (generalized) linear model ([9](#_ENREF_9)).

***Random Survival Forest (RSF):***

RSF is an ensemble tree algorithm developed to work on right-censored data. Breiman's random forest approach for survival data is extended in the Random Forests for Survival model ([10](#_ENREF_10)).

***Gradient Boosting (GB)***

GB like RSF is an ensemble method and uses base learner with CoxPH loss function. GB constructs an additive model in a forward stage-by-stage approach, optimizing differentiable loss function. ([11](#_ENREF_11)).





**Figure 1.** Heat map of 95% confidence interval of C-indices for each model of the combination of preprocessing methods, features selection, and machine learning algorithms (504 models in total).





**Figure 2.** Heat map of mean±SD of C-indices for each model of the combination of preprocessing methods, features selection, and machine learning algorithms (504 models in total).


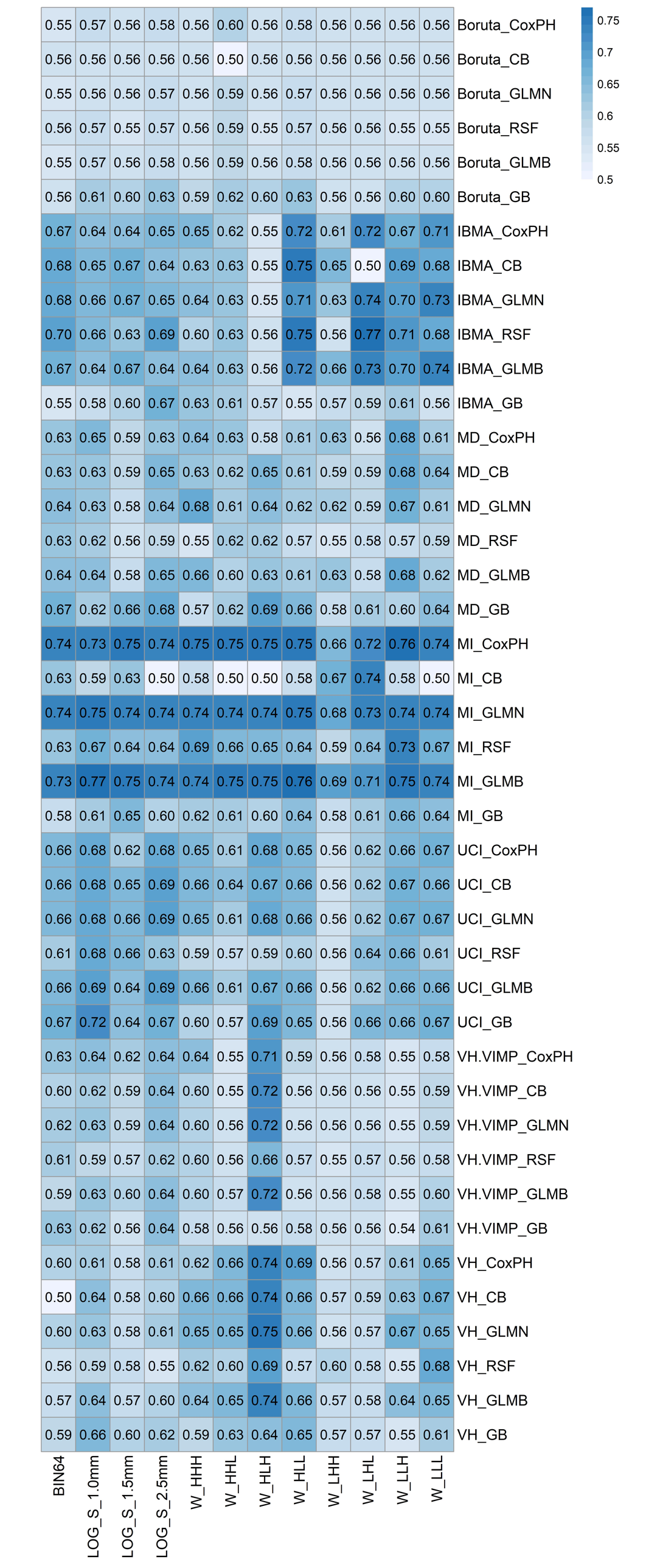


**Figure 3**. Heat map of C-indices for each model combining preprocessing methods, features selection, and machine learning algorithms (504 models in total) with 5-fold cross-validation for hyperparameter optimization.

**Figure 4**. Heat map of C-indices for each model combining preprocessing methods, features selection, and machine learning algorithms (504 models in total) with 5-fold cross-validation for hyperparameter optimization.

**
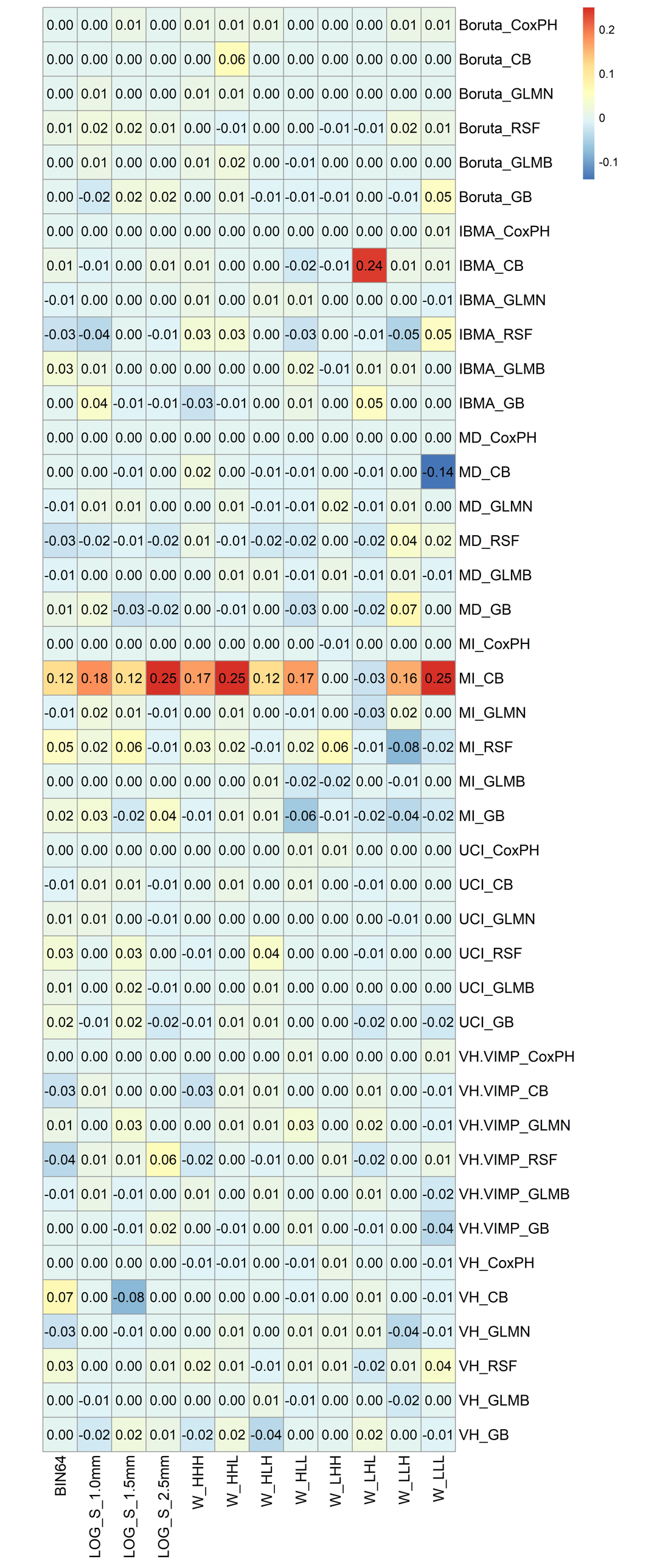
**

**Figure 4.** Difference heat map of 3-fold cross-validation to 5-fold cross-validation C-indices for each model.

**References:**

1. Ishwaran H, Kogalur UB, Gorodeski EZ, Minn AJ, Lauer MS. High-dimensional variable selection for survival data. Journal of the American Statistical Association. 2010; 105(489):205-17.

2. Ishwaran H, Kogalur UB, Chen X, Minn AJ. Random survival forests for high‐dimensional data. Statistical Analysis and Data Mining: The ASA Data Science Journal. 2011; 4(1):115-32.

3. Kursa MB, Rudnicki WR. Feature Selection with the Boruta Package. J Stat Softw. 2010; 36(11):13.

4. De Jay N, Papillon-Cavanagh S, Olsen C, El-Hachem N, Bontempi G, Haibe-Kains B. mRMRe: an R package for parallelized mRMR ensemble feature selection. Bioinformatics. 2013; 29(18):2365-8.

5. Annest A, Bumgarner RE, Raftery AE, Yeung KY. Iterative bayesian model averaging: A method for the application of survival analysis to high-dimensional microarray data. BMC bioinformatics. 2009; 10(1):1-17.

6. Fox J, Weisberg S. Cox proportional-hazards regression for survival data. An R and S-PLUS companion to applied regression. 2002; 2002.

7. Binder H, Allignol A, Schumacher M, Beyersmann J. Boosting for high-dimensional time-to-event data with competing risks. Bioinformatics. 2009; 25(7):890-6.

8. Hastie T, Qian J. Glmnet vignette. Retrieved June. 2014; 9(2016):1-30.

9. Hothorn T, Bühlmann P, Kneib T, Schmid M, Hofner B. Model-based boosting 2.0. The Journal of Machine Learning Research. 2010; 11:2109-13.

10. Ishwaran H, Kogalur UB, Blackstone EH, Lauer MS. Random survival forests. The annals of applied statistics. 2008; 2(3):841-60.

11. Chen Y, Jia Z, Mercola D, Xie X. A gradient boosting algorithm for survival analysis via direct optimization of concordance index. Computational and mathematical methods in medicine. 2013; 2013.
